# Use of black vulture (*Coragyps atratus*) in complementary and alternative therapies for cancer in Colombia: A qualitative study

**DOI:** 10.1186/1746-4269-8-20

**Published:** 2012-05-31

**Authors:** Ricardo Sánchez-Pedraza, Magda R Gamba-Rincón, Andrés L González-Rangel

**Affiliations:** 1 Department of Medicine, National University of Colombia, Bogotá, DC, Colombia; 2 Department of Human Nutrition, National University of Colombia, Bogotá, DC, Colombia; 3 Clinical Research Group, National Cancer Institute, Ave 1 # 9 – 85, Bogotá, DC, Colombia

**Keywords:** Complementary therapies, Neoplasms, Qualitative research, Falconiformes, Zootherapy

## Abstract

**Background:**

Although *Coragyps atratus* has been used as a traditional therapy for patients with cancer, the scientific literature does not contain enough information on how this therapy is used or the mechanisms that explain this therapeutic practice.

**Objectives:**

To understand the methods of use and the reasons given by patients and caregivers for the use of *Coragyps atratus* in cancer treatment.

**Methods:**

This study used a qualitative design based on twenty in-depth interviews of patients with cancer or caregivers of patients with the disease. The analysis of the text was based on an inductive thematic approach.

**Results:**

Resistance to disease and immune enhancement are properties attributed to *Coragyps atratus* when used for cancer treatment. The most recommended method of use is fresh blood ingestion, and the associated mechanism of action is transfer of immune factors to the individual who consumes it.

**Conclusions:**

Use of *Coragyps atratus* as a treatment for cancer is a popular alternative therapy in Colombia. More studies are needed to understand the clinical effects of this intervention in cancer patients.

**Spanish abstract:**

**Introducción:**

Aunque *Coragyps atratus* se usa tradicionalmente como terapia para pacientes con cáncer, no existe suficiente información en la literatura científica sobre su forma de utilización ni sobre los mecanismos explicativos que subyacen a esta práctica terapéutica.

**Objetivos:**

Conocer métodos de utilización y mecanismos explicativos dados por los pacientes y cuidadores de pacientes sobre el uso de *Coragyps atratus* en el tratamiento del cáncer.

**Materiales y métodos:**

Diseño cualitativo basado en veinte entrevistas en profundidad de pacientes con cáncer o cuidadores de pacientes con esta enfermedad. Análisis de texto basado en enfoque temático inductivo.

**Resultados:**

Al *Coragyps atratus* se le atribuyen propiedades de resistencia y fortalecimiento del sistema inmune de personas enfermas de cáncer. La forma de utilización mas común es la ingesta de la sangre fresca y el mecanismo de acción asociado es la transferencia de defensas a quien lo consume.

**Conclusiones:**

La utilización del *Coragyps atratus* como tratamiento para el cáncer es una terapia alternativa usada popularmente en Colombia. El uso de este animal debe estudiarse más a fondo para conocer los efectos clínicos en los pacientes con cáncer.

## Background

**Figure 1:**
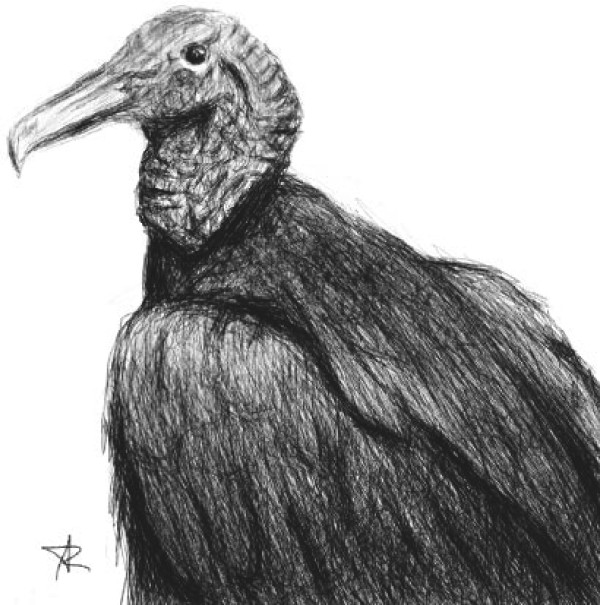
**American Black Vulture.** American Black Vulture (*Coragyps atratus*). Illustration by RSP.

The American Black Vulture (*Coragyps atratus*) is a species of scavenger bird first described by Johann Matthäus Bechstein in 1793, which is widely distributed throughout the Americas (Figure 1). In Colombia, *Coragyps atratus* is commonly called “chulo”, “gallinazo”, “zamuro”, or “golero”. These birds are generally found in open and semi-open areas and are most numerous in the outskirts of cities, primarily in garbage dumps or landfills [1]. Although *Coragyps atratus* is primarily a scavenger bird, it also attacks young and small animals [2].

Use of the Black Vulture in medicine dates back to the Roman Empire, when it was mentioned as a health treatment in texts such as *Naturalis Historia*, written by naturalist and philosopher Pliny the Elder [3]. Currently, this species has not been widely studied in basic and clinical research on human health [4]. However, in the area of complementary and alternative medicines (a group of medical systems and health care that is not considered part of conventional medicine, is not systematically taught in medical schools, and is not available in official health care systems [5]), the use of the American Black Vulture has been reported for the treatment of cancer patients. In a prevalence study performed at the Instituto Nacional de Cancerología (National Cancer Institute) in Colombia, 73.5% of 264 people surveyed reported to have used some type of complementary and alternative medicine for cancer treatment, and 2.2% of those surveyed had used *Coragyps atratus* for treating the disease [6]. In international literature, articles on the use of the American Black Vulture or other similar species in cancer are scarce [7,8]. Ethnomedical literature, however, has many examples of use of animal products for cancer treatment. Among the animals with anticancer attributes mentioned are *Boa constrictorBos indicusBos taurus*, Brachyura spp., *Bufo* sp., *Capra hircusCapra mambricaCathartes auraCaudisona durissaChelonia mydasCnemidophorus ocelliferCoendou prehensilisColomesus psittacusCrassostrea rhizophoraeCrotalus atroxCrotalus durissusCrotophaga sulcirostrisEchinaster echinophorusEunectes murinusGeococcyx californianusHippocampus reidiInia geoffrensisKerodon rupestrisLeptodactylus labyrinthicusMelipona scutellarisMephitis macrouraOreaster reticulatusPeriplaneta americanaRhinella jimiRhinella schneideriSotalia fluviatilis, Sotalia guianensisSpilotes pullatus* and *Trichechus manatus*[8-16]. Use of the Black Vulture in alternative and complementary medicine for cancer has also been described in non-scientific publications, such as local newspaper reports [17-19]. To date, we have not found studies that elucidate the use of this particular bird as therapy for patients with cancer and the explanatory mechanisms that lead to this practice.

## Methods

This study is part of two research projects on the prevalence of the use of complementary and alternative therapies in cancer management at the Instituto Nacional de Cancerología (National Cancer Institute) in Bogotá, one with adults and the other with pediatric population. Both projects were approved by the Institute’s ethics committee. A qualitative approach was used for this study, utilizing an in-depth interview method. Potential participants for the study were identified from the results of a prevalence survey performed between 2008 and 2010 (359 adults with a cancer diagnosis and 303 caregivers of children with a cancer diagnosis). Every subject who mentioned the use of the Black Vulture as a treatment was invited to participate in an interview that posed the following two questions regarding the use of this therapy: “In what way is the American Black Vulture used for treating cancer?” and “How does this type of treatment work?”

Participants who agreed to take part in the interview were asked for permission to record the interview. None of the participants objected to this type of documentation. The interviewer was a clinician with experience in this methodology, and note taking was allowed during the sessions to document content and processes that could not be registered on the recordings and could affect the interpretation of the content (for example, emotionally tinged responses). Both transcriptions of the interview recordings and notes taken by the interviewer during the sessions were used as analysis material; an inductive thematic approach was used to ensure that the themes and concepts were generated from the actual material [20]. After eighteen interviews, the information found was considered redundant. Taking into account the criteria for saturation [21], two additional interviews were conducted, which completed a sample set of twenty participants. To facilitate the organization and coding of the material, the software ATLAS.ti version 5.0 was used for managing the qualitative data. This software allows computer assisted qualitative data analysis using several tools: a code manager for simplifying the process of coding, sorting and detection of the most frequent codes in the text; a query tool for combining different codes and code families, and a network editor for visualizing connections and relationships between codes. Themes emerge as an outcome of coding, categorization and analytic reflection. Transcriptions of the interviews and interviewer’s notes were read independently by two researchers responsible for the analysis. After the initial reading, a meeting was held between the analysts and the researcher who conducted the interviews to clarify questions regarding the material that was read. Each of the analysts then independently performed a pre-codification of the categories. In another meeting between the analysts and the interviewer, a consensus was reached on the final coding categories. Finally, these categories were grouped by relevant themes that responded to the questions posed by the study. An attempt was made to reflect the veracity and validity of the data through the use of iterative procedures (sampling, initial reading of the material, pre-codification, codification, and generation of themes) and with the literal transcription of fragments of the material provided by the participants.

## Results

The group of interviewed participants contained eleven women (55%). Most participants had been diagnosed with cancer of the hematopoietic system (Table 1).

**Table 1:** Type of neoplasms diagnosed to participants

**Type of cancer**	**Frequency**	**Percentage**
Leukemia	7	35
Lymphoma	4	20
Long bone malignancy	4	20
Retinoblastoma	2	10
Hypernephroma	1	5
Ovarian	1	5
Lung	1	5
Total	20	100

For each of the identified categories, commentaries, synthesis, and transcriptions are presented that attempt to support the ultimate interpretation of the data. In some instances, the transcriptions were edited to facilitate the reading and to focus on the fundamental points. The symbols “/…/” are used in some transcriptions to indicate that the points previous to the symbol and following the symbol come from different blocks of the transcription but correspond to the same account. For each quote, the corresponding participant number is provided.

Results are grouped into three categories according to the coding that were used for the transcriptions.

### Special characteristics attributed to *Coragyps atratus*

The American Black Vulture is regarded as having special qualities, one of which is the capacity to ingest carrion without being affected, which suggests that the animal has a special strength, particularly concerning its immune system. As stated by participants: “Since the vulture eats rubbish and does not get sick, well it must have strong immune defenses/…/since it eats everything while it is decomposing, it must have good immune defenses so that it does not get sick” (participant 04)^a^; “Since they live among the garbage and do not get sick, they have good immune defenses and a lot of red blood cells and antibodies” (participant 13).^b^ Additionally, independently of the immune defense system attributed to it, the bird is regarded as an especially hardy animal that can survive adverse conditions; thus, one of the participants said: “They brought the vulture to me from the plain, it lasted several days in the house, because my son was here getting chemotherapy, the animal lasted like eight days without eating” (participant 02).^c^ In some cases, the animals are exposed to human-induced treatments to further strengthen their immune system and prepare them for use as therapy against cancer, as one patient relates: “There is a man on the coast that catches them and feeds them with meat from tumor-affected people” (participant 04).^d^

### Forms of use

Drinking the bird’s blood is the most common description of the animal’s use for cancer treatment. In this sense, having a living animal is important for extracting and drinking the blood (preferably fresh). This is illustrated by the participants: “There is a man who catches the vulture and brings it to us, he comes to the house and prepares it, so that the person can drink it right away. There he kills it and he gives you the warm blood, all of the blood that he gets from it. Not much more than a little cup of it comes out, the vulture does not have much blood. The man charges about 70000 (Authors note: amount in COP, approximately 35 USD) to catch the vulture and prepare the entire remedy” (participant 03)^e^; “With the animal alive you have to slit its throat and get the blood out, but not much comes out/…/the vulture is killed and it’s taken right away so that it does not coagulate, a little cup of it is collected” (participant 05).^f^ According to another description, the bird is prepared in the following way: “The vultures are ordered in the Mondoñedo garbage dump, or sometimes a relative brought them from Melgar. They brought them alive, and when we had them here, Grandfather hung them head down so that all the blood would run to the head, then he took everything off the neck including the feathers and he cut them in the part where the vein is, and a little cup of blood was collected, almost nothing comes out” (participant 13).^g^ Also, the importance that the animal be treated well before being sacrificed was emphasized; in this regard, a participant noted: “The vulture is taken, without hurting it, the vein is cut and the blood is extracted, half of a small cup is collected and is mixed with wine” (participant 16).^h^

In the majority of the accounts, the participants indicated that the blood is drunk in small quantities and combined with other substances, especially wines or berry juices. The objective of the mixture is not only to mask the taste of the blood but also to add substances that can reinforce the effect of the treatment: “this is how it must be given: a small cup of blood is mixed with communion wine” (participant 19)^i^; “a little cup of blood mixed with a glass of berry juice/…/the blood is the best and it is drunk mixed with berry juice” (participant 04).^j^

In addition to drinking the blood, another administration method consists of ingesting the cooked meat along with the bouillon or broth made from cooking the bird. Some descriptions that illustrate this form of treatment are as follows: “… a broth is prepared daily with each piece of the animal, the broth is made from the vulture and the person must drink all the broth and try to eat one piece every day. The person can eat whatever they can or want to, it is very good” (participant 03)^k^; “I know that one mother gave it to her son without him knowing, she gave him the meat in a soup as if it were chicken” (participant 04),^l^ and “Then the rest of the vulture was cleaned and cut up like a chicken, and a piece was prepared daily” (participant 13).^m^ Using this same method of administration, a concentrate is prepared by cooking it. The following descriptions illustrate this method of preparation: “A broth is prepared until the meat falls off the vulture’s bones, the broth is given to the child” (participant 17)^n^ and “It is cooked for a long time until all of the meat falls off the bone, about a glass-full is left” (participant 06).^o^

Although the blood of the bird is generally considered to be the most important element in the treatment, other methods of preparation of the animal are used to facilitate its administration. The other methods assume that all of the components of the bird are of equal use. In this respect, a participant commented: “The next day, the other vulture was grilled until it became very dry, it was ground up, and the powder was added to the food that she ate” (participant 15).^p^ Another interviewee mentioned that “… the bones are toasted and dried and a flour is made and beverages are made from it” (participant 17).^q^ This form of preparation appears to have been commercialized: “… a powder of dried vulture was bought and a tablespoon of the powder was put in juices” (participant 20).^r^

The last form of using the bird involves baths. The water that is left after washing the animal is used; this form of use is illustrated in the following story: “After extracting the blood the vulture is washed in hot water to pluck it, the child is bathed with this water and then wrapped in a white sheet so that he sweats, the sweating helps remove the illness” (participant 08).^s^

Regarding the dose that should be used, the strategy recommended most frequently was found to be daily administration of the treatment. Consistency was observed in the indication that the treatment should continue daily for nine days or nine weeks (the latter likely requires the use of ground and powder preparations). Regarding this duration of treatment, some participants mentioned the following: “The idea is to take the treatment for nine weeks but that is difficult because you have to get an animal for each time” (participant 04),^t^ “it is given for nine mornings which means nine vultures” (participant 07),^u^ “they say at least nine vultures should be given, but I only gave him two vultures because it is difficult to catch them” (participant 10),^v^ and “They gave the girl nine vultures for nine days, that is how it should be given” (participant 19).^w^

Regarding the timing of administration relative to the course of the illness, nothing specific was found. Only one participant reported that the treatment should be given after the administration of chemotherapy. Another element incorporated in the treatment is increasing the body temperature. In this sense, one of the subjects said: “a tablespoon of blood was extracted from the vulture which was given mixed with berry juice. After that the girl was told to run around so that she warmed up and made the treatment work” (participant 20).^x^

### Mechanism of action

The mechanism of action attributed to this treatment relates to the transfer of immune defenses. Regarding this mechanism, some participants said: “The fact that this animal eats everything gives immune defenses to people” (participant 07),^y^ “They say that vulture blood is curative or that it greatly boosts the immune defenses of people with cancer because it is assumed that since it lives among garbage and does not get sick, it has good immune defenses and a lot of red blood cells and antibodies, and that the blood helps the person create immune defenses that attack cancer” (participant 13),^z^ “by being a scavenger bird many immune defenses are created, and that means that when the person eats it, strong immune defenses are created and he or she becomes immune” (participant 15),^aa^ and “I think that this remedy acts on the immune defenses, since it is a scavenger animal and eats everything while it is decomposing, it must have strong immune defenses so that it does not get sick. And I think that it helps attack the tumor so that it does not continue to grow and it helps to create more immune defenses/…/it helps attack the tumor and bolsters the immune defenses and it does the same thing as chemotherapy. After I gave her this remedy, they did not have to give her any more chemotherapy” (participant 16).^bb^ Some of the participants referred to the immune defenses as consisting of microorganisms that attack the tumor: “This remedy is good because they say that it has microbes that attack the bad cells and bolster the boy’s immune defenses, that helps him heal” (participant 17).^cc^ Psychological explanations are not incorporated in the mechanisms of action –such as those that could be related to a placebo effect- because the efficacy of the treatment does not depend on the patient knowing that he is receiving the treatment; one comment that serves to illustrate this point is the following: “I know a mother who gave it to her son without him knowing, she gave him the meat in soup as if it were chicken and the son did not realize that she was giving him the treatment and he was cured of the cancer. He had cancer in one of his testicles” (participant 04).^dd^

## Discussion

This study attempted to obtain information regarding the manner in which a group of patients use *Coragyps atratus* as a treatment for cancer, how they administer this therapy, and the explanatory theories for the mechanism of action of the treatment. A large proportion of the findings coincide with what has been described in non-scientific literature, including the attributed properties, the preparation and the role of increasing body temperature during the administration [17-19]. Furthermore, use of other animal products in addition to blood coincide with those described in studies made with different vultures [8].

In the use of *Coragyps atratus* as a therapy against cancer, an ample gamut of administration methods was found. Among this variety in the methods of use, the ingestion of blood seems to have the greatest relevance as a therapeutic approach. We believe that the importance given to this approach is related to the belief systems rooted in different Western and pre-Columbian cultures. The importance of the blood as a container of elements that can produce an illness such as cancer or even determine temperament has been highlighted in some studies that explore beliefs around transfusions [22]. In the XVIII century in Europe, human blood, preferably consumed warm, was thought to have important therapeutic utility [23]. In some pre-Columbian American cultures, the blood possessed a strength so powerful that it could not be touched by anyone other than priests, who offered it to the gods in ritual sacrifices [24]. The results indicate that the consumption of *Coragyps atratus* in preparations such as bouillon, broth, or a cream that includes pieces of the bird are generally administered in conjunction with the ingestion of the blood. In accordance with psychoanalytical approaches, ingesting the body parts of another being transfers the qualities and powers of the devoured to the devourer [25]. The other methods of administration of *Coragyps atratus* can be associated with the need to prolong the period of treatment: the recommended period of use was found to be nine days or nine weeks, which requires the collection of a large number of birds that are not easily acquired and implicates a high cost for the patient and caregivers. In consequence, systems of commercialization of these products have been developed and seem to exist in the form of powder preparations, which last longer and can be more easily administrated than other preparations.

The participants did not offer information that allowed for an explanation of why the duration of the treatment is associated with the number nine (nine days or nine weeks). This number seems to have a mystical meaning and it is present in witchcraft and shamanic rituals as well as traditional medicine practices from other cultures [26,27]. It is believed that using a treatment for nine days grants therapeutic continuity, prevents development of tolerance to the substance and avoids occurrence of either toxicity or side effects [28]. Powers attributed to the number nine can be related to its odd nature (it is believed that an odd number of administrations results in a positive result because every even dose counteracts the previous one) and its religious attachments (nine is associated with the holy trinity, in many catholic traditions prayers are made during nine days, which is called the “novena”, a term also found in descriptions of ethnomedical practices in Spain) [26,28].

Another element that was discovered in this investigation was the practice of raising body temperature as a strategy of reinforcing the treatment with *Coragyps atratus*. The use of heat as an element has been introduced in other therapeutic inventions, with jackets, the ingestion of hot drinks [29], or as in the custom of “sweating a cold” [30], and is supported by the explanation that in some biological systems, the increase in temperature is related to efficient control mechanisms of primarily infectious processes [31]. Another explanatory theory regarding the need for this practice is reported in non-scientific literature, in which it is related to avoiding the coagulation of blood in the stomach of the person that consumes it [19]. In this study, the mechanism of action found to be attributed to the therapy is related to the transfer of factors that reinforce immunity. The importance of immune processes has been established in studies that evaluate the mechanism of action of certain herbal therapies for example, such as *Curcuma longa* and *Panax ginseng,* which appear to have immune-modulating effects [32]. Some studies have explored patient beliefs about factors that can be related to the appearance of cancer and have found that approximately 60% of those evaluated believe that immunological factors intervene in the production of the disease [33].

Many recommendations for the Colombian health system are derived from this study’s results. First, awareness of zootherapeutic practices and, in general, use of complementary and alternative medicines must be increased. Further studies are needed to evaluate the effects of black vulture’s blood in neoplastic cells *in vitro*, as has been done with other plant and animal based remedies [34]. In addition, clinical outcomes of *Coragyps atratus* use must be evaluated in cancer patients, to determine its pharmacological properties and identify possible health hazards resulting from these types of practices, especially when animal derivatives are commercialized without adequate quality control. One study has reported the presence of high levels of toxic metals in plasma and stool samples obtained from *Coragyps atratus*[35].

Increased awareness on complementary therapies must also involve the clinician-patient dyad. Some studies have proposed that official healthcare systems are based on an exclusionary logic (only one type of medicine works), while patients display a proclivity to accept the use of combined strategies in an additional or simultaneous way (complementary systems) [36]. In this study, it can be seen that patients not only combine therapies of the official systems with the administration of *Coragyps atratus*, but also incorporate other complementary and alternative medicine such as increasing body temperature and administering red fruit juices, the later believed to be useful in controlling anemia or inhibiting tumors [6,37]. We believe that it is important that subsequent studies analyze the attitude of healthcare personnel towards the option of combining official healthcare systems with complementary and alternative systems. Although not expected to incorporate zootherapy in their practice standards, physicians should ask their patients about use of complementary treatments in order to provide relevant safety information as well as being capable of detect potential complications such as toxicity and drug interactions.

One limitation of this study that should be noted, given the qualitative nature of the design and the sampling strategy used, is that the generalizability of the results is limited; this limitation implies that further studies should be conducted using a larger number of patients and specific sampling designs to draw conclusions with higher external validity. Although the majority of cases corresponded to cancer of the hematopoietic system, it cannot be suggested that this type of patient uses *Coragyps atratus* more frequently as a treatment for his or her illness than other patients.

## Conclusions

Use of *Coragyps atratus* as a treatment for cancer is a popular alternative therapy in Colombia. The most common way of use is fresh blood ingestion, and its attributed mechanism of action is transfer of immune defenses to the person who consumes it. Many cultural factors are involved in this practice, most of them linked to the belief systems rooted in different Western and pre-Columbian cultures.

## Endnotes

^a^ “Como el chulo come porquerías y no se enferma, pues debe tener muchas defensas/…/como come todo en descomposición debe tener buenas defensas para que no se enferme”.

^b^ “Como viven en la basura y no se enferman, tienen buenas defensas y muchos glóbulos rojos es decir anticuerpos”.

^c^ “Me trajeron el chulo de los llanos, duró varios días en la casa, como mi hijo estaba recibiendo quimioterapia el animal duró como ocho días sin comer nada”.

^d^ “Hay un señor en la costa que los agarra y los cría con carne de enfermos, con tumores”.

^e^ “El chulo nos lo trae un señor que los coge y viene hasta la casa de uno y lo prepara, para que la persona lo consuma de una vez. Allí lo mata y le da la sangre caliente, toda la sangre que se le extrae ya que no sale más de una copadita, el chulo tiene poquita sangre. El señor cobra como 70.000 por agarrar el chulo y hacer todo el remedio”.

^f^ “Estando vivo el animal hay que degollarlo y sacarle la sangre que no sale mucha/…/se mata el chulo y se da una vez para que no se coagule, se recoge un copadita”.

^g^ “los chulos son encargados en el botadero de Mondoñedo, o a veces nos los traía un familiar de Melgar. Los traían vivos, ya cuando los teníamos aquí, el abuelo los colgaba con la cabeza hacia debajo de tal manera que la sangre se le viniera toda hacia la cabeza, luego le quitaba todo, las plumas del pescuezo y le cortaba en la parte de la vena, se recogía una copadita de sangre, ya que casi no sale”.

^h^ “Se coge el chulo, sin lastimarlo, se le corta la vena y se le extrae la sangre, se recoge media copadita la cual se mezcla con vino”.

^i^ “esta es la forma como se debe dar: se le da una copadita de la sangre mezclado con vino de consagrar”.

^j^ “una copadita de sangre que se mezcla en un vaso de jugo de mora/…/la sangre es lo mejor y se toma revueltica con jugo de mora”.

^k^ “… le preparaba un caldo diario con cada presa del animal, se hace el caldo con el chulo y la persona debe tomarse todo el caldo y tratar de comerse un presa diaria. Se puede comer todo lo que la persona pueda y quiera, es muy bueno”.

^l^ “Yo sé que una mamá se lo dio al hijo a escondidas, le dio la carne entre la sopa como si fuera un pollo”.

^m^ “Luego el resto de chulo era arreglado y despresado como una gallina, y se le preparaba diariamente una presa”.

^n^ “Se prepara un caldo hasta que se deslía el chulo que solo quede el hueso, el caldo se le da al niño”.

^o^ “Se cocina mucho tiempo hasta que se deslía todo el chulo, queda como un vaso”.

^p^ “Al otro día, el otro chulo se asó hasta que quedó bien seco, se trituró, y se le agregaba de este polvo a todas las comidas que ella ingería”.

^q^ “… los huesos se tuestan y se secan y se hace la harina y con ella se hacen coladas”.

^r^ “… se le compraba el polvo de chulo seco y se le daba una cucharada de este polvo en los jugos”.

^s^ “luego de extraer la sangre se lava el chulo en agua caliente para desplumarlo, con esa agua se baña al niño y se envuelve en una sábana blanca, para que se sude, ese sudor le ayuda a sacar la enfermedad”.

^t^ “La idea es tomar el tratamiento por nueve semanas pero es difícil porque para cada vez hay que conseguir un animal”.

^u^ “se le da por nueve mañanas es decir nueve chulos”.

^v^ dicen que se deben dar por lo menos nueve chulos, pero yo solo le di 2 chulos, porque es difícil agarrarlos”.

^w^ “A la niña se le dieron nueve chulos por nueve días, esa es la forma como se debe dar”.

^x^ “se le extraía una cucharada de sangre del chulo, la cual se le daba en jugo de mora. Después de eso se ponía a trotar a la niña para que se caliente y le haga el tratamiento”.

^y^ “El hecho de que este animal coma de todo le da defensas a las personas”.

^z^ “Dicen que la sangre del chulo pude curar o le sube mucho las defensas a las personas con cáncer, porque se supone que como viven en la basura y no se enferman tienen buenas defensas y muchos glóbulos rojos es decir anticuerpos y esa sangre hace que la persona cree defensas que atacan el cáncer”.

^aa^ “por ser una ave de rapiña crean muchas defensas, y esto hace que la persona al comerlo cree defensas y se inmunice”.

^bb^ “Creo que este remedio actúa en las defensas, como es un animal carroñero, y come todo en descomposición debe tener buenas defensas para que no se enferme. Y creo que le ayuda a atacar el tumor para que no siga creciendo y ayuda a que cree más defensas/…/ayuda a atacar el tumor y sube las defensas y hace lo mismo que la quimioterapia, después que yo le di ese remedio no tuvieron que hacerle más quimioterapias”.

^cc^ “Este remedio es bueno porque dicen que tiene unos microbios que atacan las células malas y suben las defensas del niño, eso ayuda a que se sane”.

^dd^ “Yo sé que una mamá se lo dio al hijo a escondidas, le dio la carne entre la sopa como si fuera un pollo, el hijo no se dio cuenta que le estaban dando el tratamiento y se curó del cáncer, tenía cáncer en un testículo”.

## Competing interests

The authors declare that they have no competing interests.

## Authors' contributions

RSP conceived of the study, participated in its design and coordination and helped to draft the manuscript. MGR and AGR incorporated background references, helped with the discussion and completed the manuscript. All authors read and approved the final manuscript.
